# Toward a Clinically Reliable Class II Resin Composite Restoration: A Cross-Sectional Study into the Current Clinical Practice among Dentists in Saudi Arabia

**DOI:** 10.1155/2022/2691376

**Published:** 2022-08-02

**Authors:** Rasha AlSheikh, Khalid S. Almulhim, Moamen Abdulkader, Rasha Haridy, Amr S. Bugshan, Rand Aldamanhouri, Moataz Elgezawi

**Affiliations:** ^1^Department of Restorative Dental Sciences, College of Dentistry, Imam Abdulrahman Bin Faisal University, Dammam, Saudi Arabia; ^2^Department of Substitutive Dental Sciences, College of Dentistry, Imam Abdulrahman Bin Faisal University, Dammam, Saudi Arabia; ^3^Department of Clinical Dental Sciences, College of Dentistry, Princess Nourah Bint Abdul Rahman University, P.O. Box 84428, Riyadh 11671, Saudi Arabia; ^4^Department of Conservative Dentistry, Faculty of Dentistry, Cairo University, Cairo, Egypt; ^5^Department of Biomedical Dental Sciences, College of Dentistry, Imam Abdulrahman Bin Faisal University, Dammam, Saudi Arabia; ^6^Internship Program, College of Dentistry, Imam Abdulrahman Bin Faisal University, Dammam, Saudi Arabia

## Abstract

**Objective:**

The aim of this study was to evaluate the current clinical practice of general dentists in Saudi Arabia in restoring class II cavities using direct resin composites and to set evidence-based practice recommendations of concern.

**Methods:**

An online survey formed of 20 questions and classified into four domains was developed. 500 dentists in 5 Saudi provinces were invited to join the survey anonymously and voluntarily using poster announcements and e-mail invitations. Descriptive statistics were used to analyze participants' responses.

**Results:**

343 responses were received. Dentists in Saudi Arabia vary in their clinical practices and techniques of insertion of resin composite in class II cavities. 67% of participants use cotton rolls for isolating the field while 32% use rubber dam isolation. 33% and 28% of respondents use circumferential matrix (Tofflemire) and AutoMatrix, respectively. Fracture, followed by recurrent caries and open proximal contacts, was the received main reason of failure of class II direct resin composite restorations.

**Conclusion:**

Diversity of class II resin composite practices exists among dentists in Saudi Arabia. For ensuring optimum quality outcomes and high standards of restorative dentistry healthcare, several dentists in Saudi Arabia need to reconsider their clinical practice and modify their clinical procedures of direct class II resin composites. Several evidence-based practice guidelines are recommended to dentists in this article to improve their practice and enhance the clinical reliability and longevity of class II direct resin composite restorations.

## 1. Introduction

Direct resin composites are the first choice and most widely used direct restoration of posterior teeth with a good survival rate and reported annual failure rate of 1–3% [1]. Long-term clinical reliability of a biomechanically and esthetically satisfactory restoration is a core focus of interest of dentists and patients [2]. Ongoing advancement in resin composite materials, adhesive technologies, and light curing devices and application methodologies has been coupled with increased patients' knowledge and expectations of restoring teeth with optimum esthetic qualities, established biocompatibility, and superior functional effectiveness at a reasonable cost with shortest possible time needed for fulfilment of the restoration. Nevertheless, resin composite has limitations and demerits that need thoughtful consideration during its use. Polymerization shrinkage, technique sensitivity, difficulty in building up effective proximal contacts, and long-term [3] biodegradation [4] are among the reported clinical challenges of resin composite restorations that necessitate meticulous attention to all clinical procedures of cavity preparation and resin composite application [5] as well as effectively performing the postoperative care [6] and maintenance [7, 8].

Several resin composite materials have been recently introduced to the market to improve clinical performance and patient satisfaction [9–11]. Bulk-fill, self-adhesive, antibacterial, and self-healing resin composites are among the modern formulations. Innovations in resin composite organic matrix, filler technology, coupling agents, initiator system, and tinting agents have enabled fabricating esthetically superior and bifunctionally remarkable quality resin composite restoration even in posterior teeth where esthetics might be considered of less clinical significance than in anterior teeth [12–16].

Among all hard tooth lesions, class II cavities present a demanding clinical situation and require adequate knowledge and clinical skills [17]. Restoring tight proximal contacts has been extensively reported among the challenges of class II direct resin composite restorations [18–21]. On the other hand, adaptation at the cervical margins of class II resin composites, where inadequacy or lack of enamel for effective etching is frequently encountered, is highly demanding and obligating considerate procedures.

Several application techniques and matrix systems were suggested to ensure tight proximal contacts and allow restoring physiological contours and embrasures [22] which is mandatory for maintaining healthy gingival and periodontal tissues and avoiding wedging of food between teeth [23, 24].

Among the most famous matrix systems are the sectional matrix, circumferential matrix, and AutoMatrix systems. Among the clinical insertion techniques, incremental insertion, bulk-fill insertion, and firstly building a proximal wall of composite have been widely used. Moreover, the use of a flowable liner of resin composite has been advocated among the recommended techniques of insertion [8]. On the other hand, several light curing techniques have been suggested to adequately cure resin composite, particularly in class II situations where adequately curing the deepest zone of resin composite away from the light curing tip is questionable. In addition to occlusal light curing, three-directional curing, use of light reflecting wedges, and bulk-fill composites have been suggested.

The FDI Commission Project 2–95 has outlined three factors influencing the quality of dental restorations. These included material related factors which are related to each restorative material's properties, merits, and demerits. Furthermore, patient related factors including the socioeconomical factors, the specific nature of the oral environmental conditions, and the oral hygienic status were reported as significant factors influencing the effectiveness and longevity of dental restorations. Nevertheless, it reported the operator related factors as fundamental factors in determining the quality and clinical performance of dental restorations. These included procedural excellence variations, judgement, and perceptual variations among operators in different aspects comprising cavity design, material selection, and manipulation [25].

Delivery of evidence-based patient-centered oral and dental healthcare has been a fundamental standard of all healthcare bodies and distinguished medical and dental educational and treatment centers that seek to excel and perfect the offered clinical dental services [26–28]. Therefore, this study was designed to analyze the current clinical practice of dentists in Saudi Arabia in class II resin composite restorations and to set several recommendations to improve this practice.

## 2. Materials and Methods

An online survey of 20 questions divided into four domains was developed. The targeted population was 500 dentists appointed in general governmental hospitals in 5 Saudi provinces, middle, north, west, east, and south. The three largest governmental hospitals in each of the included Saudi cities, according to the number of available dental clinics in each, were selected. University and private hospitals and dental centers were not included in the study. To test the consistency and validity of the questions, ten senior professors and/or consultants of restorative dentistry were invited to evaluate the study questionnaire categorizing respective answers as irrelevant, of low relevance, relevant, or highly relevant. All experts' responses were relevant or highly relevant to all questions. Poster announcements were provided in the selected hospitals inviting dentists to join the online survey. Furthermore, e-mail addresses of the targeted dentists were collected anonymously through personal contacts without revealing any of their personal data. E-mail invitations were sent to them to join the online survey. Invitations briefly described the survey objectives and mentioned the affiliating institutes. Participants were assured that joining the survey is voluntary and anonymous and they are free to skip any question or leave the survey at any time. A contact e-mail was available in the invitation for any inquiry concerning the survey.

An ethical approval of Imam Abdulrahman Bin Faisal University IRB Committee was obtained before starting the survey. Descriptive statistics were used to analyze the obtained responses.

## 3. Results

Table 1 summarizes the domains, questions, and respective responses of participants in numbers and percentages for each respective answer option.

The greatest participation was obtained from east, west, and south provinces. There was a lack of general agreement between participants regarding their usual practice or main procedure followed during resin composite insertion. 37% of respondents were 31–39 years old while 28% were 23–30 years old. On the other hand, 12% were above 50 years old and 23% between 40 and 49 years old. Most of the responders were male taking up 68% of the total surveys, and 32% were female. The participants were mainly from the eastern province, accounting for 37% of the participants, followed by the western and southern province, each accounting for 22%. The northern and middle province had the least numbers of surveys with 10% and 8%, respectively.

The total years of practice of dentistry varied between the responders; 33% of the responders have been practicing for more than 15 years, while 24% have been practicing for less than 5 years. Another 24% have been practicing for 5–10 years, and 19% for 10–15 years. 41% of the responders perform less than 10 new class II composite restorations on average per week, and 39% perform more than 10 restorations, while 20% perform more than 20 restorations. A large portion (54%) of the responders repair or remake less than 5 faulty class II composite restorations on average per week. On the other hand, 26% repair more than 10, and 20% repair more than 5 restorations per week.

Based on the responder's personal procedural preferences, the most popular (67%) choice of isolation used in class II composite restoration was cotton rolls while 32% use rubber dam isolation. The remaining 1% of the responders did not use any form of isolation. The types of composites used by the responders for class II were micro-hybrid (42%) and nano-hybrid (39%). 4% used other types of composites, while 14% did not know what type of composite they use. 55% used the conventional incremental technique for restoring class II, while 37% used the flowable bulk-fill followed by conventional composite, and 8% used bulk-fill only. The light curing method mostly used (86%) was light emission diode (LED), and 4% used the quarts. 10% of the responders did not know what type of light curing method they use. The responders mainly used three-directional curing method in their class II with a percentage of 72%, and the remaining 27% used the occlusal direction. 87% of the responders chose not to heat their resin composite before insertion, while only 12% chose to heat it. 69% of the responders answered that they started by a layer of flowable composite on the gingival seat before progressing to the successive resin composite increments; on the other hand, 30% did not start with a flowable composite. The adhesive systems used for class II resin composite by the responders were etch-and-rinse (51%), selective etch (15%), and self-etch technique (14%). The rest of the responders, accounting for 19%, use any available system they have. The most popular types of matrix systems used for restoring class II with resin composite by the responders were Tofflemire, sectional matrix, and AutoMatrix with percentages of 33%, 29%, and 28%, respectively. Less commonly used matrix systems were Triodent V3 matrix (7%) and FenderMate matrix (2%). 77% have answered that they first built a proximal wall of composite against the matrix before proceeding to subsequent increment of composite, while 21% did not.

Based on the responders' feedback about old composites, 51% claimed that, upon using floss to test the proximal contacts, they were satisfied with the techniques they use for producing tight contacts, and 17% were very satisfied. However, 23% answered with “kind of” and 7% with “not at all” satisfied. 36% of the responders did not take bitewings to check the quality of class II, while 35% did sometimes, and 28% always did. The most common failures of class II encountered by the responders in their clinic that necessitate remake or repair were fracture 32%, recurrent caries (26%), pain (19%), open contact (17%), and discoloration (6%). When asked about the incidence of post-restorative hypersensitivity reported by their patients in the first week after class II restoration, 46% responded with “rare” and 32% with “infrequent.” However, 19% said “sometimes,” and 3% said they frequently encountered reported incidence of postoperative hypersensitivity.

## 4. Discussion

Evidence-based clinical practice confirms the clinical reliability of class II direct resin composite restorations. Extensive systematic reviews, meta-analyses, clinical trials, and observations that generally employ modified USPHC and/or FDI criteria of clinically ranking restorations [29–31] have been used to document the long-term effectiveness of these restorations [1]. In a recent clinical trial, resin composite class II restorations showed excellent performance with no need for repair or remake after 24-month interval using the FDI criteria, with evident patients' satisfaction irrespective of scarce cases of mild hypersensitivity and less tight proximal contact. In another clinical study, class II resin composites performed well regardless of the resin composite material [32].

Dentists related factors that can influence the quality of outcomes of class II resin composite restorations are extensive and cover variations in all restorative management steps and restorative treatment decisions. In all clinical situations, it is the responsibility of the dentist to undergo proper diagnosis and execute appropriate cavity preparation design features. Furthermore, he/she should be able to select the most suitable resin composite material and adhesive approach and employ effective light curing procedures as well as sound resin composite insertion techniques. Moreover, he/she should ensure adequate finishing and polishing methods [8, 25, 33].

The scope of current cross-sectional study outlines the clinical practices and procedures followed by dentists in Saudi Arabia during restoring class II cavities using direct resin composites. This encompasses variations in their preferred resin composite materials, light curing methods, insertion techniques, and finishing and polishing procedures. Critical factors in rubber dam isolation, adhesive bonding approaches, light curing devices, insertion techniques, and matrix systems are principally addressed.

The obtained responses of the first domain of demographic factors showed that most of participants were less than 40 years of age, which reflects the significance of the survey topics to young dental practitioners. The greatest contribution of dentists was from Eastern and Western provinces as dentists there might have been encouraged by the academic institutes to which investigators are affiliated and their wider and more convenient advertisement of the survey in participating hospitals. The finding that the percentage of participation of dentists decreases with greater number of new class II resin composite weekly restorations and with the increased clinical years of experience is in line with a pervious similar survey performed in England [25]. This might be related to the greater number of younger generation and less experienced dentists appointed in the selected hospitals. Moreover, it might reflect higher level of satisfaction of more experienced dental practitioners with their current practice of class II resin composites. Additionally, it can indicate their broader awareness of the different complicated and challenging aspects of class II resin composites as well as the different procedural protocols.

Among the main concerns of restorative dentistry practice is failure of restorations. It has been reported that 50% of resin composite restorations fail in 10 years. It has been estimated that about 60% of restorative dentistry practice time on average is spent in repair or remake of faulty resin composites [34]. In a novel 11-year observation period retrospective study, the one-year failure rate of resin composite in posterior teeth treated by undergraduate students, indicated by the need for re-intervention, was 5.73%. At five years, it was 16.7%, while in ten-year period, it was 18.74% [35].

The received answers of respondents of this study confirm that remake and/or repair of faulty class II resin composites constitute a regular part of clinical practices of class II resin composites, which necessitates adequate analysis of the potential reasons of failure and setting a number of recommendations for improving the standard of restorative dental healthcare [35].

Our findings that 67% of participants use cotton rolls for field isolation, while 23% use rubber dam isolation, and 1% do not isolate the field during resin composite insertion in class II cavities raise concerns. The importance of rubber dam isolation during restoring teeth with resin composite is well documented. A former clinical study reported that at 10 years of clinical service, the behavior of restorations of posterior teeth with resin composite placed under well-controlled, effective isolation with cotton rolls and aspiration was not significantly different from that of restorations placed using rubber dam isolation. However, this same study argued that the use of high suction aspiration in combination with cotton rolls was essential [36]. Considerable modern evidence confirmed the importance of rubber dam isolation for high-quality class II resin composite restorations [37]. While many reports indicate that proper rubber dam isolation is mandatory for a high-quality resin composite restoration [8], others recommended using it whenever possible [30].

In a previous survey studying the clinical practices of posterior resin composites among general dental practitioners in England, 68% (173 dentists) of the respondents reported that they never use rubber dam while 94% declared they routinely use cotton roll isolation. In another survey in Dental Practice-Based Research Network, 64% of the participating dentists reported that they do not use rubber dam with any restoration [38].

A basic aspect in the practice of resin composite restorations is the selection of effective, durable, and clinically reliable resin adhesive bonding approach. Etch-and-rinse approach is considered as a gold standard approach where phosphoric acid is used to etch both enamel and dentin parts of the preparation before water rinsing and dryness prior to the application of a bonding resin. Alternatively, self-etch resin bonding approach, which is easier and less time consuming, facilitates synchronized etching and resin impregnation of the dentin collagen which might confirm complete penetration of the exposed collagen layer of dentin [7]. To make bonding to enamel more effective, selective etching is used to firstly etch enamel parts of the preparation using phosphoric acid before implementing self-etching approach. Our findings that 51% of responding dentists use the etch-and-rinse approach while 14% use self-etch approach, indicate that most of dentists in Saudi Arabia are attentive to the evidence-based recommendations. On the other hand, the 19% of respondents who may use any available adhesive boding technique in class II resin composites might need to reconsider their protocol [9, 31].

The declaration made by 86% of dentists taking part in the current survey about using LED blue light curing for curing resin composites in class II cavities, versus 4% using Quartz Tungsten Halogen light and 10% who reported they did not know the type of light curing they use, is interesting. While it fits the literature data indicating that most dentists around the world employ LED light as a satisfactory curing light for curing resin composites [39], it highlights the significance of confirming the awareness among dentists in Saudi Arabia of the significance of the employed curing light.

Our findings that 42% and 39% of respondents use micro-hybrid and nano-hybrid resin composite materials in class II cavities, respectively, indicate that most dentists in Saudi Arabia are in line with previous general recommendations of a meta-analysis for improving the quality of resin composite direct class II restorations [31]. At the same time, the 4% and 14% of respondents that either use another category of resin composite or do not know the kind of resin composite they use for restoring class II cavities need to improve awareness and modify their clinical practice.

The current survey data showing that 55% and 37% of participants use the conventional incremental technique of insertion and the insertion of an initial flowable resin composite increment while 8% use bulk-fill composite, are different from those obtained from a previous similar study. This previous study was done in only one area of Saudi Arabia where the participating dentists reported horizontal and oblique layering as the mere methods of insertion of resin composite in posterior teeth [40].

The collected feedback that, upon insertion of resin composite in class II cavities, 69% use an initial layer of flowable composite conforms with previous recommendations aiming to increase the adaptation at the critical cervical margin of class II cavities [3–5, 41]. Moreover, the 87% of participating dentists who do not preheat resin composite before insertion are not consistent with the findings that preheating resin composite reduces marginal gaps [42].

The received responses that 72% of dentists perform three-directional curing occlusally, buccally, and lingually while 27% use occlusal light curing, reflect the awareness among the majority of dentists in Saudi Arabia. This agrees with evidence highlighting the significance of the depth of curing light penetration, cavity configuration, and intervening tooth substance for the adequacy of light curing and the degree of conversion for the clinical performance of class II resin composite [39].

A recent study reviewed the available in vitro and in vivo evidence to set a best strategy for insertion of resin composites in class II cavities. Incemental insertion, bulk insertion, firstly inserting a flowable layer, and preheating of composite were among the searched techniques. The study showed that the literature does not recommend a specific insertion technique of resin composite in class II cavities. Furthermore, it has drawn a clinical significance, indicating that regardless of the technique of insertion employed, appropriate and meticulous attention during insertion and light curing of resin composite has the most significant influence on the quality of the final class II resin composite restoration [43, 44].

Our results that 33%, 28%, 29%, 7%, and 2% of participants use circumferential matrix, AutoMatrix, sectional matrix, or its modified forms of Triodent V3 or FenderMate systems, respectively, might raise the importance of drawing dentists' attention to the effectiveness of the employed matrix system. To ensure a tight proximal contact of class II resin composite restorations, the use of sectional matrix has been advocated by many in vitro and in vivo investigations [9, 45].

The finding that 69% of participants firstly build a wall of resin composite against the used matrix before progressing to subsequent increment insertion of resin composite is riveting since it is in harmony with many recent recommendations for ensuring effective, tight, and physiological proximal contact. Nevertheless, it demonstrates the importance of enhancing the awareness of 21% of dentists in Saudi Arabia who do not use it [6].

The frequency of forms of failure of class II resin composite restorations encountered by the participating dentists of the current study is in line with previous similar surveys in different locations around the world [1, 30]. It is captivating that 46% and 32% of responses reported that incidence of post-restoration hypersensitivity in the first week after restoring class II cavities with direct resin composite was rare and infrequent, respectively. This might reflect that most of dentists in Saudi Arabia follow adequate protocols in management of class II lesions restored with direct resin composites including careful diagnosis, selection of materials, cavity preparation, and resin composite insertion techniques. Findings about material insertion indicate that many dentists in Saudi Arabia pay meticulous attention during the insertion and curing of direct resin composite in class II cavities to control the adverse consequences of polymerization shrinkage of resin composite where post-restoration hypersensitivity comes first in the list [2–4, 7, 46, 47]. On the other hand, it appears that the 19% and 3% of participants who reported first week post-restoration hypersensitivity in 20% and more than 50% of patients after class II resin composite restorations need to start corresponding appropriate practice modification.

This study was a cross-sectional study that was conducted over a period of four months, from August to December 2020. During that period the worldwide COVID-19 pandemic evolved with all the restrictions that affected the clinical practice as well as dental education [46, 48]. This might have affected the size of the targeted population at the time of study design as well as the level of respondents to the survey. A greater population size and higher level of respondents could have provided a broader study outcome. Another limitation of the current study is related to the included general governmental hospitals that grant dental treatments to patients completely free of charge. This can have an influence on the practice relative to the amount of flow of patients seeking free dental treatments versus time and facilities availability. Adding private dental centers and clinics as well as university clinics and hospitals to the study might have influenced the study outcomes and included general dentists exposed to socioeconomically different patients and distinctive facilities.

## 5. Conclusions

Under the circumstances of the current investigation and considering all its limitations, the following conclusions could be drawn:

1. Dentists in Saudi Arabia are not consistently following the same protocol of directly inserting resin composites in class II cavities including materials selection, field isolation, insertion technique, light curing, and matrix system.
2. While many dentists in Saudi Arabia use satisfactory evidence-based practices, some need to reconsider their clinical practice and protocols followed during resin composite insertion in class II cavities.

## Figures and Tables

**Table 1:** Survey questions, responses, and percentages.

Questions	Modalities	Number of responses (%)	Total number of responses (%)
(i) Demographic aspects			
(1) Age	23–30	97 (28%)	343 (100%)
	31–39	126 (37%)	
	40–49	79 (23%)	
	Above 50	41 (12%)	
(2) Gender	Male	233 (68%)	343 (100%)
	Female	110 (32%)	
(3) Where have you been practicing in Saudi Arabia?	Eastern province	127 (37%)	338 (99%)
	Western province	77 (22%)	
	Middle province	27 (8%)	
	Northern province	33 (10%)	
	Southern province	74 (22%)	
(4) How long have you been practicing dentistry?	Less than 5 years	82 (24%)	342 (100%)
	5–10 years	81 (24%)	
	10–15 years	66 (19%)	
	More than 15	113 (33%)	
(ii) Practice frequency			
(5) How many new class II resin composite restorations on average do you perform per week?	Less than 10 restorations	140 (41%)	342 (100%)
	More than 10 restorations	134 (39%)	
	More than 20 restorations	68 (20%)	
(iii) Personal procedural preferences			
(6) What is the kind of isolation that you use during class II resin composite restoration? (More than one answer could be selected)	Cotton rolls	230 (67%)	343 (100%)
	Rubber dam isolation	112 (32%)	
	No isolation	1 (1%)	
(7) What is the type of resin composite material that you usually use for making class II restorations?	Micro-hybrid	143 (42%)	340 (99%)
	Nano-hybrid	136 (39%)	
	Other (please specify)	14 (4%)	
	I do not know	47 (14%)	
(8) What is the insertion technique that you follow in restoring class II cavities?	Conventional incremental technique	187 (55%)	342 (100%)
	Bulk-fill technique	29 (8%)	
	Flowable bulk-fill followed by conventional composite	126 (37%)	
(9) What is the light curing unit that you usually use?	Light emission diode (LED)	294 (86%)	247 (99%)
	Quartz Tungsten Halogen (QTH)	14 (4%)	
	I do not know	34 (10%)	
(10) What is the light curing direction that you use for curing resin composite in class II cavities?	Occlusal direction	93 (27%)	341 (99%)
	Three-directional curing (occlusal, buccal, lingual)	248 (72%)	
(11) Do you usually preheat resin composite before insertion?	Yes	43 (12%)	341 (99%)
	No	298 (87%)	
(12) Do you usually start by a layer of flowable composite on the gingival seat before progressing to the successive resin composite increment?	Yes	237 (69%)	340 (99%)
	No	103 (30%)	
(13) What is the type of adhesive system that you use for restoring class II cavities with resin composite?	Etch-and-rinse	174 (51%)	341 (99%)
	Selective etching technique	53 (15%)	
	Self-etch technique	50 (14%)	
	Any available system	64 (19%)	
(14) What is the type of matrix system that you use for restoring class II cavities with resin composite?	Circumferential matrix–retainer device	114 (33%)	338 (98%)
	Sectional matrix	100 (29%)	
	Triodent V3 matrix	23 (7%)	
	FenderMate matrix	5 (2%)	
	AutoMatrix	96 (28%)	
(15) In restoring class II cavities with resin composite, do you first build a proximal wall of composite against the matrix before proceeding to the subsequent increment of composite?	Yes	265 (77%)	338 (98%)
	No	73 (21%)	
(iv) Post-restorative quality hypersensitivity and tightness of proximal contact			
(16) How satisfied are you with the techniques that you use for producing tight plus proximal contacts upon testing proximal contacts using dental floss?	Not at all	23 (7%)	337 (98%)
	Kind of	80 (23%)	
	Satisfied	176 (51%)	
	Very satisfied	58 (17%)	
(17) Do you usually check the quality of your class II resin composite restorations using bitewing radiographs postoperatively?	Yes	97 (28%)	343 (100%)
	No	125 (36%)	
	Sometimes	119 (35%)	
(18) How many faulty class II resin composite restorations do you repair and/or remake per month?	Less than five	183 (54%)	342 (100%)
	More than five	69 (20%)	
	More than 10	90 (26%)	
(19) What is the main form of failure of class II resin composite you encounter in your clinical practice necessitating repair/remake?	Fracture	111 (32%)	341 (99%)
	Recurrent caries	89 (26%)	
	Open contact	59 (17%)	
	Pain	64 (19%)	
	Discoloration	20 (6%)	
(20) What is the frequency of post-restorative hypersensitivity reported by the patient in the first week after your class II resin composite restoration?	Rare (less than 2%)	158 (46%)	342 (100%)
	Infrequent (less than 10%)	109 (32%)	
	Sometimes (20% or more)	66 (19%)	
	Frequent (more than 50%)	9 (3%)	

## Data Availability

Data are available upon request.
